# Dental drugs with proarrhythmic risk in patients with Brugada syndrome: precaution instructions for practices in the field of orofacial pain

**DOI:** 10.3389/fcvm.2026.1754683

**Published:** 2026-02-04

**Authors:** Dawool Han, Na Yeong Cho, Eunae Sandra Cho, Seung-Young Roh

**Affiliations:** 1 Department of Oral Pathology, Oral Cancer Research Institute, Yonsei University College of Dentistry, Seoul, Republic of Korea; 2 BK21 FOUR Project, Yonsei University College of Dentistry, Seoul, Republic of Korea; 3 Division of Cardiology, Department of Internal Medicine, Korea University College of Medicine, Seoul, Republic of Korea

**Keywords:** Brugada syndrome, dentistry, inherited arrhythmias, neuropathic pain, orofacial pain

## Abstract

Orofacial pain, diagnosed and treated in a subfield of dentistry, highly relies on long-term psychotropic, neuromodulating, and analgesic regimens that have the potential to alter cardiac ion channels and autonomic tone. In patients with inherited arrhythmia syndromes such as Brugada syndrome (BrS), these drugs may unmask a type 1 Brugada electrocardiographic pattern or trigger malignant ventricular arrhythmias and sudden cardiac death, yet most dental guidance addresses only short-term use of local anesthetics. In this narrative review, we synthesize evidence on the arrhythmogenic potential of medications used for orofacial pain—non-steroidal anti-inflammatory drugs, tricyclic antidepressants, anticonvulsants, and primary headache therapies. Finally, we propose a pragmatic risk-stratified approach for orofacial pain specialists. Incorporating channelopathy-specific precautions into orofacial pain pharmacotherapy may reduce drug-induced ventricular arrhythmias and sudden cardiac death while preserving effective long-term pain control.

## 1 Introduction

Orofacial Pain is a specialty in Dentistry that covers the diagnosis and treatment of non-odontogenic craniofacial and oral pain disorders, including masticatory and musculoskeletal pains, joint diseases of the jaw, headaches, neuralgias, neuropathies, sleep disorders, and psychosocial problems related to orofacial pain (1). As many orofacial pain disorders are chronic in nature, long-term medication treatment use is common compared to other subfields in Dentistry. Psychotropic, neuromodulating medications and muscle relaxants are regular therapeutics for orofacial pain, regardless of whether the patient has accompanying psychiatric disorders. Although anxiety and depression have been frequently reported in patients with chronic orofacial pain, psychotropic and neuromodulating medications are specifically prescribed for the resolution of chronic pain itself rather than for the alleviation of psychiatric symptoms (2–5).

Pharmacological management and cautions are well recognized and updated for dental treatment practice, including those for cardiac patients with arrhythmia and channelopathies (6–9). Despite the academic community's effort, most of these reports and instructions are limited to local dental surgery or conventional odontogenic treatment focused on local anesthesia and short-term antibiotic usage. In this review, we will discuss cautions and safety instructions for dental clinicians when considering orofacial pain medications in cardiac arrhythmia patients with a focus on Brugada syndrome(BrS). This review covers analgesics, tricyclic antidepressants, anticonvulsants, and medications aimed at primary headaches that have the potential to aggravate BrS or evoke BrS-like symptoms.

## 2 Cardiac arrhythmias and brugada syndrome

Arrhythmias are aberrant cardiac rhythm disorders that may be or may not be symptomatic (10). Lethal arrhythmias may result in sudden cardiac death(SCD), even in patients who lack predisposing clinical symptoms or problematic electrocardiogram (ECG) signals in everyday settings (11, 12).

Despite the difficulties of predicting fatal arrhythmic occurrence in healthy individuals, predisposing channelopathies are known as proarrhythmic risk factors in patients with structurally normal hearts. This tendency is more pronounced in individuals under the age of 35, with arrhythmia being the main cause of SCD despite the absence of cardiac structural abnormalities and other symptomatic systemic diseases (13, 14). The low incidence of SCD in this age population indicates that hereditary risk factors such as channelopathies may serve as a major cause of lethal arrhythmia-induced SCD in patients without structural cardiac disorders (14–16). In total, inherited arrhythmia syndromes account for about 2%–10% of the causes of SCD, being more prevalent in Asia compared to the other continents (17–20).

In this review, we will focus mainly on one of the most prevalent inherited arrhythmias, BrS (20, 21). All four of these hereditary channelopathies are known to induce fatal arrhythmias by inherited mutation(s) in their cardiac ion channels (21, 22). Cardiac ion channels control physiological heart rhythm by regulating the action potential in cardiac cells (23). Inherited or acquired mutations in cardiac ion channels may induce aberrant rhythms, inducing arrhythmia (24). Channelopathies encompass all disorders regarding ion channels, such as autoimmune channelopathies (25), yet in this article, the term “channelopathies” is limited to mutation-associated channelopathies to focus on the pathophysiology of inherited arrhythmias.

BrS is an inherited arrhythmia syndrome and channelopathy, most commonly showing gene mutation in the SCN5A gene, which encodes the cardiac voltage-gated sodium channel *α* type V (26). The initial report in 1992 described 8 normally structured cardiac patients with a characteristic coved-type ST elevation and ventricular fibrillation leading to a high risk for SCD (27). The epidemiologic prevalence of BrS is 1 per 2,000 (28). BrS has a higher incidence in males and in the Asian race, specifically in the Southeast Asia region (14, 29–31). BrS is estimated to induce about 4% of the total SCDs, while the numbers increase up to 20% when considering the incidence of SCDs in patients with normally structured hearts (32).

As BrS patients may be asymptomatic (31, 33), a known family history or diagnosis of BrS may be the only evidence presented to the patient's dentist at their dental appointment. Recommendations have been reported for BrS patients in the dental clinic, yet the instructions are limited to routine minor surgeries or dental interventions with local anesthesia use (8, 9, 34–36). Awareness has been raised for local dental anesthetics use in BrS patients, but lacks scientific evidence, including routine dental anesthetics such as 2% lidocaine with 1:100,000 epinephrine (8, 37). The Brugadadrugs.org Advisory Board classifies lidocaine as class IIb (There is conflicting evidence and/or divergence of opinion about the drug, and the potential arrhythmic effect in BrS patients is less well established by evidence/opinion) and “preferably avoided in BrS patients” with a comment that clarifies local lidocaine injections to be considered safe for dental practices when combined with 1:100,000 epinephrine (38, 39). Other than lidocaine and procaine (yet another local anesthetic used in dentistry), there is minimal information on other medications used in the dental clinic, especially drugs that are used as long-term therapeutics, as frequently prescribed in the Orofacial Pain field.

## 3 Orofacial pain medication use in brugada syndrome

Table 1 (39, 40) is a list of medications advised to be “avoided” or “preferably avoided” in BrS that are commonly used in the field of Orofacial Pain. The following text describes some of the most representative medications in Table 1 in detail.

(1) Non-steroidal analgesics

**Table 1:** List of orofacial pain medications to avoid in Brugada syndrome.

Generic name	Clinical use	Use in orofacial pain	Pathophysiology	Evidence base	References
Amitriptyline	TCA	Neuropathic pain Myofascial pain Insomnia Migraine Chronic tension HA Fibromyalgia	Blocks sodium channels, widens QRS complex	Narrative reviews (UK/Malaysia), retrospective case series (*n* = 402, USA), case reports (Netherlands, Japan), experimental studies (*in vitro*)	(65–69)
Carbamazepine	Anti-convulsant	Trigeminal neuralgia Neuropathic pain Post-herpetic neuralgia	Blocks neuronal and cardiac sodium channels	*In vitro* patch-clamp study (Netherlands), case-control study (*n* = 5,473 cases, *n* = 21,866 controls, Netherlands), retrospective cohort (Netherlands), case reports (Oman, Japan)	(73, 75–77, 80)
Lamotrigine	Anti-convulsant	Trigeminal neuralgia Dysesthesia Neuropathic pain	Shares binding sites in neuronal sodium channels	*In vitro* patch-clamp studies, case reports (Italy, USA, Portugal)	(73, 81–84)
Nortriptyline	TCA	Neuropathic pain Myofascial pain Temporomandibular joint disorder Migraine Idiopathic facial pain	Blocks sodium channels, widens QRS complex	Same evidence base as amitriptyline	(65–69)
NSAIDs	Analgesic Anti-inflammatory	Acute pain Inflammatory pain Migraine Tension-type HA	Target cardiac channels hNav1.5 (SCN5A), hKv11.1 (KCNH2), and TRPM7	*In vitro* studies, mechanistic studies, genetic studies	(51–54)
Oxcarbazepine	Anti-convulsant	Trigeminal neuralgia Neuropathic pain SUNCT/SUNA	Structural derivative of carbamazepine—shares binding sites in neuronal sodium channels, cardiac sodium channel blockage	*In vitro* patch-clamp mechanistic study (same as carbamazepine—structural derivative)	(73)
Phenytoin	Anti-convulsant	Trigeminal neuralgia Neuropathic pain Migraine	Shares common anticonvulsant binding site in neuronal Na + channels, cardiac sodium channel blockade	*In vitro* binding site study	(73)
Sumatriptan	Anti-migraine	Migraine Cluster HA	Potential sodium channel blocker	Case reports (Japan, USA)	(86–88)
Tramadol	Narcotic analgesic	Severe pain in orofacial region	Sodium channel blockadge	International expert consensus/literature review (http://www.brugadadrugs.org, multi-country collaboration), cross-sectional study (*n* = 1,402 patients, Iran)	(39, 94)
Verapamil	Calcium-channel blocker Anti-hypertensive	Cluster HA Migraine	Calcium-channel blocker	Experimental study, case reports (Turkey, Japan), mechanistic studies	(89–92)

HA, Headache; NSAIDs, Nonsteroidal anti-inflammatory drugs; SUNCT/SUNA, Short-lasting unilateral neuralgiform headache with conjunctival injection and tearing/Short-lasting unilateral neuralgiform headache with cranial autonomic symptoms; TCA: Tricyclic antidepressants.

The field of orofacial pain has a strong foundation in chronic pain, yet diagnosis and treatment cover all stages. Distinguishing between acute and chronic pain is ambiguous, as it usually relies on the patient's statement of pain initiation. Chronic pain may be defined as more than 3 months of continuous pain, but assessing the presence of neuroplastic factors, such as central sensitization, may be more helpful when choosing treatment strategies for the individual patient (41–43).

In definite acute pain or nociceptive pain with an inflammatory origin, non-steroidal anti-inflammatory drugs(NSAIDs) are first in line (3). NSAIDs are widely used before and after dental/orofacial interventions, in craniofacial traumas and orofacial inflammations (44, 45). NSAIDs, specifically selective cyclooxygenase(COX)-2 inhibitors, are known to have cardiovascular side effects such as pro-thrombotic activity and blood pressure increase that higher the risk of myocardial infarction, strokes, hypertension, heart failure, arrhythmias, and sudden cardiac death (46–48). This led to a decline in selective COX-2 inhibitor NSAID use in clinics. Certain studies have argued that non-selective NSAIDs, such as naproxen, have a cardioprotective effect, while other studies reported certain COX-related cardiac effects in non-selective NSAIDs as well (46, 47).

Other than COX-related cardiac effects, NSAIDs in general have the potential to perturb physiologic ion channel activities. NSAIDs that target channels have their therapeutic benefits as blocking certain ion channels on neuronal cells or immune cells may induce pain or inflammation alleviation (49, 50). Certain NSAIDs, such as, target cardiac channels such as human sodium channel hNav1.5, potassium channel hKv11.1, and cation channel-kinase TRPM7, which are encoded by the gene SCN5A, KCNH2, and TRPM7, relatively (51, 52). SCN5A is one of the most common mutated genes in BrS, and there are reports of KCNH2 mutations in symptomatic BrS (53, 54). Nevertheless, most of the studies are on a pre-clinical level, lacking large-population or case-controlled clinical evidence. Unlike sodium channel blockers (flecainide, propafenone) that are antiarrhythmic drugs labeled “to be avoided in BrS” by the BrugadaDrugs.org Advisory Board, there aren't any formal instructions for whether NSAID use is safe in BrS patients. For fever control in patients with a general arrhythmic risk, some clinical guidelines favor acetaminophen over NSAIDs due to the increased risk of atrial fibrillation when using NSAIDs, but this is scientifically uncertain in BrS (55–58). Fever itself can induce BrS patterns and increase the risk of SCD in BrS patients; thus, clinicians should not hesitate to use active anti-pyretic therapeutics in these situations, while closely monitoring the patient when using NSAIDs (36, 59–61).

(2) Tricyclic antidepressants

Tricyclic antidepressants (TCAs) are known to be effective in chronic orofacial pain that doesn't have an inflammatory origin. TCAs and anticonvulsants modulate neuropathic pain and musculoskeletal disorders at a central nervous system level with lower doses than when used for typical depression (3, 62, 63). For myofascial pain syndrome, burning mouth syndrome, and chronic temporomandibular disorders, Amitriptyline and nortriptyline are prescribed first in line at a daily dose of 10–50 mg for at least a month to be effective, while in depression, the initial and maximum dosage is much higher, up to 150 mg/day if needed (3, 64).

TCAs can induce blockade of sodium channels, which widens the QRS complex (the most prominent part of an ECG, which represents rapid depolarization and contraction of the ventricles) and can unmask a Brugada-type pattern (65–69). Reviews and case series document coved ST-segment elevation and ventricular arrhythmias more frequently with overdose, also known as TCA poisoning. Although not every patient develops ECG changes, the potential to trigger a diagnostic type 1 pattern in latent BrS has been reported. As TCAs are used in lower doses in the dental clinic, it is logical to assume that the risk of cardiac side effects is lower in orofacial pain patients. Nonetheless, authoritative avoidlists categorize several TCAs, including amitriptyline and nortriptyline, as drugs to avoid or preferably avoid in BrS (39). Thus, when considering therapeutics for chronic orofacial pain, TCA alternatives should be considered as when BrS is known or suspected, and clinicians should consider baseline ECG in patients with syncope, family history of sudden cardiac death, or prior abnormal tracings before initiating these agents.

(3) Anticonvulsants

Anticonvulsants are used for neuralgias and neuropathies in orofacial pain, most frequently in trigeminal neuralgia. Carbamazepine is first in line for trigeminal neuralgia. Oxcarbazepine and phenytoin are alternatives for carbamazepine-unresponsive trigeminal neuralgias (70–72). Carbamazepine, oxcarbazepine (a structural derivative of carbamazepine), phenytoin, and other anticonvulsants share binding sites in neuronal sodium channels (73). These anticonvulsants inhibit neuronal firing induced by normal, light touches by perturbing sodium currents that over-excite neuronal cells (72, 74). Carbamazepine alone or combined with other anticonvulsants increases the risk for cardiac sodium channel blockage, aberrant heart rhythms, and cardiac arrest (75–77).

The BrugadaDrugs.org Advisory Board recommends oxcarbazepine as “drugs to be avoided” and carbamazepine and phenytoin as “drugs to preferably avoid” (39). In BrS patients with refractory neuralgia non-sodium-blocking alternatives should be considered. Gabapentin, an anticonvulsant used as a first-line drug for postherpetic neuralgia and post-traumatic trigeminal neuropathic pain, and second-line drug for myofascial pain syndrome, burning mouth syndrome, and trigeminal neuralgia, is currently not on the list of drugs to avoid in BrS (3). Gabapentin, which doesn't directly affect voltage-gated sodium and potassium channels, binds to calcium channel subunits to regulate neural activity involved with chronic pain and seizure (78). Unlike calcium channel blockers, such as verapamil (mentioned below), gabapentin does not directly block the channel pore, and a study reported that it did not alter the electrophysiological activity in cardiac channels *in vitro* (79). Nevertheless, gabapentin can theoretically regulate the activity of cardiac L-type calcium channels, a mutation target spot in BrS, and requires population-based clinical evidence to be assumed as 'safe' for BrS.

If carbamazepine is unavoidable, low starting doses, careful titration, and electrocardiographic surveillance are prudent. Since anticonvulsants have been reported to induce Brugada-type ST-segment elevation and malignant ventricular arrhythmias in certain patients (80–84), therapy should be withheld in any patient who develops a type-1 BrS pattern or unexplained syncope.

(4) Medication for primary headaches

Primary headaches such as migraine, tension-type headaches, and trigeminal autonomic cephalalgias are covered in the field of orofacial pain as well. BrS and drug-induced type 1 BrS pattern patients have a higher prevalence of migraines (85). There has been a report on BrS patterns showing in a cluster headache patient as well (86). Moreover, antimigraine drugs used for migraine and cluster headache can act as potential sodium channel blockers, evoking BrS patterns or worsening BrS symptoms. Post-sumatriptan-induced BrS has been reported frequently (86–88).

Another drug for cluster headache and migraine is verapamil, a calcium-channel blocker also used for hypertension and angina. Verapamil is classified as “to preferably avoid” by BrugadaDrugs.org (39). Verapamil modified heart rhythm patterns in experimental BrS settings, but the results weren't consistent in clinical studies (89). Verapamil toxicity may induce BrS patterns, and even normal doses of it can aggravate BrS when coexisting with vasospastic angina, raising caution when using calcium channel blockers in symptomatic BrS or BrS with concomitant cardiac disorders (90–92).

(5) Evidence synthesis and clinical application

Guidelines and consensus documents converge on several principles that are directly applicable to the orofacial pain specialist or dental clinic (32, 93). High-risk drugs should be avoided when safer alternatives are available, and electrocardiography should be obtained when baseline risk is elevated or when sodium-channel blocking agents are being considered (12, 39). Fever and electrolyte disturbances should be corrected promptly because both can precipitate arrhythmic events in BrS and in patients with limited repolarization reserve (32). Drug–drug interactions require systematic review at every visit to strong sodium-channel blockers, and to inhibitors of drug metabolism that raise psychotropic exposure (39).

In the chronic orofacial pain context, pharmacologic selection can be aligned with these principles without compromising analgesia. TCAs and carbamazepine have important analgesic roles but should be avoided in known or suspected BrS and used with ECG surveillance in others (3, 4, 62). Non-pharmacologic interventions, local therapies, and non-opioid analgesics can reduce systemic doses and mitigate risk.

A practical monitoring algorithm begins with risk screening that includes family history of sudden death, prior syncope, previous abnormal ECGs, and current medications. When risk is high or when sodium-channel blockers are considered, a baseline ECG is obtained, and electrolytes are optimized. Drug choice then favors agents with lower cardiac liability, and patient education emphasizes the need to treat fever, maintain hydration, and report palpitations or syncope. ECG is repeated when doses increase, when interacting medications are added, or when symptoms arise (12, 21, 32, 39).

## 4 Conclusions

Psychotropic and neuromodulating agents are indispensable in chronic orofacial pain, yet they modify cardiac electrophysiology through sodium-channel blockade, calcium-channel blockade, or autonomic activation. Certain NSAIDs, TCAs (amitriptyline, nortriptyline), anticonvulsants(carbamazepine, oxcarbazepine, lamotrigine, phenytoin), and primary headache medications(sumatriptan, verapamil) may induce a BrS pattern ECG or aggravate underlying BrS symptoms. Gabapentin is not currently in the “drugs to avoid in BrS” list and may be considered as an alternative drug for orofacial pain but requires further clinical-level evidence to assure safety in BrS patients. In BrS patients or those with cardiac rhythm disorders, consultation with a cardiac specialist, the use of alternatives, and periodic ECG monitoring are recommended.
